# Unveiling how vitrification affects the porcine blastocyst: clues from a transcriptomic study

**DOI:** 10.1186/s40104-021-00672-1

**Published:** 2022-03-15

**Authors:** C. Almiñana, F. Dubuisson, S. Bauersachs, E. Royer, P. Mermillod, E. Blesbois, F. Guignot

**Affiliations:** 1 UMR PRC, INRAE 0085, CNRS 7247, Université de Tours, IFCE, F, –37380 Nouzilly, France; 2 Functional Genomics Group, Institute of Veterinary Anatomy, VetSuisse Faculty Zurich, University of Zurich, Zürich, Switzerland; 3 UEPAO, INRAE, F, –37380 Nouzilly, France

**Keywords:** Blastocyst, Embryo, Gene expression, In vitro culture, Porcine, RNA–sequencing, Transcriptomics, Vitrification

## Abstract

**Background:**

Currently, there is a high demand for efficient pig embryo cryopreservation procedures in the porcine industry as well as for genetic diversity preservation and research purposes. To date, vitrification (VIT) is the most efficient method for pig embryo cryopreservation. Despite a high number of embryos survives in vitro after vitrification/warming procedures, the in vivo embryo survival rates after embryo transfer are variable among laboratories. So far, most studies have focused on cryoprotective agents and devices, while the VIT effects on porcine embryonic gene expression remained unclear. The few studies performed were based on vitrified/warmed embryos that were cultured in vitro (IVC) to allow them to re–expand. Thus, the specific alterations of VIT, IVC, and the cumulative effect of both remained unknown. To unveil the VIT-specific embryonic alterations, gene expression in VIT versus (vs.) IVC embryos was analyzed. Additionally, changes derived from both VIT and IVC vs. control embryos (CO) were analyzed to confirm the VIT embryonic alterations. Three groups of in vivo embryos at the blastocyst stage were analyzed by RNA–sequencing: (1) VIT embryos (vitrified/warmed and cultured in vitro), (2) IVC embryos and (3) CO embryos.

**Results:**

RNA–sequencing revealed three clearly different mRNA profiles for VIT, IVC and CO embryos. Comparative analysis of mRNA profiles between VIT and IVC identified 321, differentially expressed genes (DEG) (FDR < 0.006). In VIT vs. CO and IVC vs. CO, 1901 and 1519 DEG were found, respectively, with an overlap of 1045 genes. VIT-specific functional alterations were associated to response to osmotic stress, response to hormones, and developmental growth. While alterations in response to hypoxia and mitophagy were related to the sum of VIT and IVC effects.

**Conclusions:**

Our findings revealed new insights into the VIT procedure-specific alterations of embryonic gene expression by first comparing differences in VIT vs. IVC embryos and second by an integrative transcriptome analysis including in vivo control embryos. The identified VIT alterations might reflect the transcriptional signature of the embryo cryodamage but also the embryo healing process overcoming the VIT impacts. Selected validated genes were pointed as potential biomarkers that may help to improve vitrification.

**Supplementary Information:**

The online version contains supplementary material available at 10.1186/s40104-021-00672-1.

## Introduction

Currently, there is an increasing demand for reliable and practical porcine embryo cryopreservation protocols for commercial use in the porcine industry as well as for genetic diversity conservation and biomedical research purposes. Efficient embryo cryopreservation methods would indeed allow: long–term conservation, transport and widespread dispersion of high–quality genetics resources; simplify the international transportation of specific genetic backgrounds with a minimal risk of disease transmission and at reduced cost; while avoiding an important impact on animal welfare during transportation. Altogether, it would allow the porcine breeding companies to introduce their genetics with maximum health guarantees in emerging markets [1, 2], which is essential when facing eventual health crises. In addition, many local breeds are currently decreasing, progressively replaced by highly selected commercial ones. The extinction of such breeds adapted to specific environment would decrease the genetic diversity of the species and may induce the loss of alleles that could become of high interest in the future. In parallel, an increasing number of genetically engineered pigs are produced as models for studying human diseases, which also requires good cryopreservation protocols as well as better in vitro embryo culture systems [3–5].

For many years, attempts to cryopreserve pig embryos were unsuccessful compared to other species such as bovine. The substantial lipid content of porcine embryos makes them very sensitive to chilling injuries [6]. The development of the vitrification technique led to a great advance in pig embryo cryopreservation [7, 8]. Since then, many studies have tried to optimize the porcine embryo vitrification procedure by focusing on different technical factors such as the concentration of cryoprotective agents (CPAs) in the cryopreservation media, the use of different warming media, the benefits of applying chemically or semi–chemically defined media, cooling rate, cryopreservation devices, etc. [9–21]. The success of most of these vitrification systems/protocols has been determined based on the in vitro survival rate after embryo warming [12, 14, 22] and in some minor cases, based on pregnancy and farrowing rates of the vitrified embryos after embryo transfer [23, 24].

To date, vitrification is the most efficient and widely used method in pig embryo cryopreservation, particularly the ultra–rapid vitrification systems [11, 25–29]. Vitrification has been successfully used on embryos at early stages of development [14, 30] and also on oocytes [20, 31, 32]. However, the lower pregnancy rates achieved for vitrified embryos (ranging from 39% to 75%) when compared to fresh ones [24, 33], the need of highly qualified personnel for vitrification and surgical embryo transfer to achieve high pregnancy and prolificacy rates, represent major hurdles for pig embryo cryopreservation. Moreover, pregnancy and farrowing rates when using vitrified embryos are highly variable among laboratories. Results seem also to be dependent on the embryo stage or the breed [30, 34–36]. Taken together, it points to a technique that needs constant adaptations in the protocol.

On the other hand, little has been done on determining the impact of the vitrification procedure on the porcine embryo in terms of embryonic alterations at the gene expression level, which could bring valuable clues about the reasons for the embryo damage and provide hints to overcome it or to confer embryo protection. Up to date, most studies on gene expression of vitrified embryos have been performed using a targeted approach by analyzing a panel of selected genes [16, 37, 38] or based on microarray technology [39]. Here, we used a more extensive transcriptomic approach by using RNA–sequencing to analyze molecular alterations induced by vitrification. Furthermore, most studies analyzing the effect of vitrification on the embryo gene expression used vitrified/warmed embryos that are cultured in vitro for a variable period of time allowing them to recover and re–expand (6 h, 24 h, 48 h) [39–41], avoiding to include dead embryos or embryos that are not completely recovered in the gene expression analysis. This leads to the analysis of the cumulative effect of VIT and IVC, while the specific alterations induced by VIT remain unknown.

Therefore, the objective of the study was to determine the VIT procedure-specific embryonic alterations by comparing VIT versus (vs.) IVC embryonic gene expression at the blastocyst stage. Additionally, changes derived from both VIT and IVC vs. control embryos (CO) were analyzed to confirm the VIT embryonic alterations. The strategy of this study was to dissect specific effects of VIT or IVC and to select candidate biomarkers of embryo quality after vitrification. The data derived from this study was expected to provide new insights into transcriptome alterations induced by VIT and to contribute to a better understanding of processes in pig embryos after VIT and IVC procedures which can affect embryo development and fate.

## Materials and methods

### Experimental design

In vivo embryos were collected surgically. Then, embryos showing intact zona pellucida and with good or excellent quality based on their morphology appearance [42] were selected, divided in three groups and used for transcriptomic analysis as follows: VIT, subjected to vitrification (including an in vitro culture step of 24 h for embryo restructuration and expansion after warming); IVC, in vitro culture (24 h in vitro culture after embryo collection without vitrification) and CO, control embryos (not subjected to VIT or IVC procedures). The VIT protocol was based on the protocol described by Cuello et al. [11, 12] (detailed below). A total of 23 gilts were used as embryo donors and 394 embryos were recovered (blastocyst stage). Only embryos with good or excellent quality after vitrification/warming and in vitro culture were used for transcriptome analysis as detailed below.

### Materials

Chemicals were purchased from Sigma–Aldrich (Saint–Quentin Fallavier, France), unless otherwise stated.

### Animals

All animal procedures were performed under the ethical approval of the French Ministry of Agriculture (authorization number APAFIS#3923–2016020409132759. v4). Experiments were conducted at the INRAE experimental farm in Nouzilly (France). Large White hyperprolific (LWh) cyclic gilts, aged from 7 to 8 months, were used as embryo donors. They were kept individually in crates in a mechanically ventilated confinement facility, facing each other for improving their welfare. They were fed with a commercial ration of 3 kg/d. Water was provided ad libitum.

### Synchronization treatment, detection of estrus and artificial insemination

All chemical products used for embryo production were purchased from Centravet, Beaucouzé, France. For all the experiments, LWh gilts (*n* = 23) were synchronized and superovulated and used as donors. A first estrus cycle of the donors was synchronized by oral administration of Altrenogest (Regumate®), 5 mL/d during 18 d. The onset of estrus occurred 6 d after the end of Regumate administration (d 0). On d 14, two injections (10 and 16 h) of 175 μg of cloprostenol i.m. (Planate®) were performed. Superovulation was induced by injecting 1000 IU PMSG i.m. (Chronogest®) 24 h after the second cloprostenol injection. Ovulation was induced by administration of 500 IU hCG i.m. (Chorulon®) 48 h after the PMSG injection. Estrus detection was performed twice a day by exposing females to a mature boar. Usually, estrus occurred 24 to 36 h after hCG treatment. Gilts showing signs of estrus were artificially inseminated 12 and 24 h after the onset of estrus with 3 × 10^9^ spermatozoa per dose prepared with semen from adult LW boars and were referred as donors.

### Embryo collection and embryo quality assessment

Embryos were surgically recovered from donors at d 6 after ovulation induction. Donors were sedated by administration of ketamine (20 mg/kg bodyweight, i.m.) and of xylazine (2 mg/kg bodyweight, i.m.). Anesthesia was maintained by inhalation of 3% isoflurane. A midline incision was made on the white line to externalize the reproductive tract. Embryos were collected by flushing the first 40 cm of the top of each uterine horn (near utero–tubal junction) with 40 mL of TALP–HEPES containing 0.1 g/L PVA [43]. Embryos were recovered under a stereomicroscope equipped with a heating plate. Their developmental stage was examined at 60 × magnification. Only embryos at the blastocyst stage, 160 to 200 μm in diameter, with a good or excellent morphological appearance and with intact zona pellucida were selected for further experiments.

### Embryo vitrification and warming

The vitrification protocol used in the present study was previously described by Cuello et al. [11, 12]. This protocol [11, 12] was selected for the study based on the good results in terms of in vitro embryo survival and also pregnancy rates. During the entire procedure of vitrification, the temperature of media and embryos was maintained at 39 °C with a heating plate. Groups of 4 to 7 embryos were vitrified together using a 4–well multidish (Nunc 4–well culture plate; Dutscher, Issy–les–Moulineaux, France). Embryos were firstly washed twice for 5 min in TALP–HEPES containing 0.1 g/L PVA. Then, they were transferred to the same medium but complemented with 5% (v/v) ethylene glycol (EG) and 7.5% (v/v) dimethylsulfoxide (DMSO) (equilibration solution: ES). Then, embryos were incubated for 3 min in ES and then for 1 min in vitrification solution (VS, TALP–HEPES containing 0.1 g/L PVA, 16% (v/v) EG, 16% (v/v) DMSO and 0.4 mol/L sucrose). Subsequently, embryos were placed in a 1 μL droplet and then loaded together by capillarity into the narrow end of a superfine open pulled straw (SOPS, Minitube, Tiefenbach, Germany) during the VS step. Straws containing the embryos were then plunged horizontally into liquid nitrogen.

The embryo warming procedure used was based on the one–step dilution method [14]. Straws containing the embryos were removed from the liquid nitrogen and the narrow end was submerged vertically in a well of a four–well multidish containing 1000 μL TALP–HEPES containing 0.1 g/L PVA with 0.13 mol/L sucrose at 39 °C. Then, the embryos were allowed to fall by gravity into the well, rinsed in TALP–HEPES with 0.13 mol/L sucrose warming medium for 5 min, and subsequently, transferred to TALP–HEPES 0.1 g/L PVA medium without sucrose for 5 min.

### In vitro culture of embryos and assessment of in vitro embryo survival

After warming, embryos were washed for 3 min in 500 μL of NCSU–23 medium [44] containing 0.4% BSA and 10% fetal calf serum. They were cultured in 500 μL of the same medium in four well plates for 24 h under 700 μL mineral oil at 38.8 °C in a humidified atmosphere of 5% CO_2_ in air. After in vitro culture, embryos were evaluated morphologically for their developmental progression under a stereomicroscope. Vitrified blastocysts that regained the volume of their blastocoel cavities after warming and those blastocysts exhibiting a normal zona pellucida with good appearance were considered viable.

Additionally, a group of embryos showing good morphology after embryo collection were in vitro cultured for 24 h and assigned to the IVC embryo group. Embryo survival rates and hatching rates were evaluated at 24 h of in vitro culture (for embryos vitrified/warmed and for embryos only subjected to IVC). The survival rate was defined as the ratio of viable/cultured embryos. The hatching rate was defined as the ratio hatched/viable embryos.

### Embryo transfer and evaluation of pregnancy and farrowing rates

Surgical embryo transfers were performed on d 5 of the estrous cycle. For synchronization, recipients received Altrenogest (Regumate®), 5 mL/d, during 17 d and 2.5 mL the 18th day. Their ovulation was induced 4 days later by administration of 500 IU hCG i.m. (Chorulon®). The onset of estrus was detected the next day and twice daily by exposing recipients to a mature boar.

On the day of the embryo transfer, 30 cryopreserved embryos were transferred per recipient, so several straws (5 to 8) were thawed successively. Just after warming, embryos were grouped in a small drop of TALP–HEPES 0.1 g/L PVA. They were aspirated in a catheter connected to a syringe. The embryos were introduced into the upper end of one uterine horn after a mid–ventral laparotomy of a synchronized recipient.

Pregnancy was assessed around 30 and 45 d post–estrus by ultrasonography. Pregnant gilts were allowed to go to term. The number of piglets was recorded at farrowing. The survival rate at farrowing was defined as the live–born piglet/transferred embryos and expressed as a percentage.

### Transcriptomic analysis of embryos by RNA–sequencing: RNA isolation, low–input total RNA library preparation, and sequencing

Embryos subjected to VIT, IVC or CO as detailed above were used for transcriptomic analysis by RNA–sequencing. Pools of 4–6 embryos, from the three procedures, were snap frozen in liquid nitrogen and used for RNA isolation as follows: 1) VIT embryo samples (9 replicates, a total of 44 embryos in 8 pools of 5 embryos and 1 pool of 4 embryos); 2) IVC embryo samples (6 replicates, a total of 31 embryos in 5 pools of 5 embryos and 1 pool of 6 embryos); and 3) CO embryos samples (6 replicates, a total of 30 embryos in 6 pools of 5 embryos). Total RNA from these 21 embryo pool samples was isolated using the RNeasy Micro kit (QIAGEN) according to the manufacturer’s instructions. RNA quality and concentration were analyzed using the Agilent 2100 Bioanalyzer (Agilent Technologies, Santa Clara, CA, USA), NanoDrop (Thermo Fisher, Waltham, MA, USA), and Quantus Quantiflour® RNA system (Promega). Samples with best RNA quality and concentration were selected for preparation of RNA–Seq libraries (15 libraries, 5 replicates/embryo treatment). The RNA integrity number (RIN) for all embryo samples ranged between 8.50 and 10.

For library preparation, the Ovation SOLO RNA–Seq System Kit (NuGen Technologies, San Carlos, USA) was used. Library preparation followed the manufacturer’s instructions. In brief, aliquots of 1 ng of total RNA were prepared for each embryo pool sample as starting material for RNA–Seq library preparation. First, samples were subjected to DNase treatment and primer annealing, followed by cDNA processing and second strand synthesis. After end repair, adapters were ligated, and the number of PCR cycles for the first library amplification step was determined by qPCR according to the manual. To remove fragments derived from ribosomal RNAs, NuGEN’s insert–dependent adaptor cleavage (InDA–C) technology was performed in the next step. At the same time, strand selection was performed. After this step, the second library amplification and purification were performed for each sample using a universal primer and a set of barcode primers for sample multiplexing. Once RNA–seq libraries were prepared, quantitative and qualitative analyses were performed for each of the libraries using Agilent 2100 Bioanalyzer DNA High Sensitivity assays and Quantus Quantiflour® ONE dsDNA system (Promega). Sequencing of the libraries was conducted on an Illumina HiSeq 2500 instrument at the Functional Genomics Center Zurich (FGCZ). Pooled barcoded libraries were run on two lanes of a single–end flow cell generating between 4 and 11 million single–end reads (125 bp) per sample.

RNA–sequencing data analysis was performed as described recently in Bauersachs et al. [45]. Briefly, the obtained sequence reads (Fastq files) were analyzed with an established analysis pipeline integrated in a local Galaxy installation [46] at the Animal Physiology group, ETH, Zurich. Processing, quality control, mapping, and quantification of the obtained sequences was performed as previously described [45]. The porcine genome assembly ARS–UCD1.2 (SusScrof11.1) was used and the corresponding GFF3 annotation file from NCBI. Based on mapping information for the reads (BAM files processed with NuDup to remove PCR duplicates), a read count table for all annotated porcine genes was generated using QuasR Qcount. This count table was filtered to remove sequences with negligible read counts by using counts per million (CPM) per sample filtering tool [47]. The mean library size and potential CPM cutoff (Counttable statistics, custom Galaxy tool) was calculated and the cutoff set to 10 CPM (corresponding to an average of 15 reads per library) for at least 3 out of 15 libraries. This count table was the basis for the subsequent statistical analysis.

The analysis of differential gene expression was performed using BioConductor package EdgeR [48]. Data normalization was performed on library size (TMM normalization) [49] and with the GLM robust (estimateGLMRobustDisp) [50] function. For comparison of the experimental groups, the following contrasts were set: VIT vs. CO; IVC vs. CO and VIT vs. IVC. An adjusted *P*–value (false discovery rate, FDR) of 0.001% was used as threshold for significance of differential gene expression for VIT vs. CO. Because of the lower number of DEGs obtained for the other two comparisons, the likelihood ratio (LR) of 13.80 corresponding to FDR 0.0012% in the IVC vs. CO comparison and to FDR 0.0058% in the VIT vs. IVC was used as a threshold for the other two comparisons to achieve a comparable stringency and sensitivity of the significance analysis [45, 51]. RNA–Seq data have been deposited at NCBI’s Sequence Read Archive (SRA) under the BioProject accession PRJNA697877 and is available at https://www.ncbi.nlm.nih.gov/sra/PRJNA697877.

### Data mining and bioinformatics analysis of RNA–seq data

Gene symbols and Entrez Gene IDs (porcine and putative human orthologs) were mapped for all transcripts, using bioinformatics custom tools integrated in a local Galaxy installation. Custom database tools (NCBI annotation mapper, Mammalian Annotation database, MADb, https://madb.ethz.ch [52]) were used to assign known or putative human orthologous genes. Human gene identifiers or symbols were used for subsequent functional annotation. To compare mRNAs altered due to VIT, IVC technologies compared to CO, or VIT vs. IVC Jvenn an integrative tool for comparing lists of genes with Venn Diagrams was used [53]. To obtain information about overrepresented biological functions Metascape tool was used [http://metascape.org] [54].

### Quantitative real–time polymerase chain reaction (qPCR) analysis

Embryonic gene expression analysis for 8 selected genes, based on RNA-seq results, was performed in all embryo groups (VIT, IVC and CO) by qPCR. The genes analyzed were: epithelial membrane protein 1 (*EMP1*), annexin A8 (*ANXA8*), glutathione S–transferase alpha 4 (*GSTA4*), plasminogen activator inhibitor 1 precursor (*SERPINE1*), placenta expressed transcript 1 (*PLET1*), BTG anti-proliferation factor 2 (*BTG2*), secreted phosphoprotein 1 (*SPP1*), and solute carrier family 10 member 1 (*SLC10A1*). The primer sequences for these genes are listed in Table 1. First, Crescendo cDNA Synthesis for qPCR kit (TECAN Sales Switzerland AG, Maennedorf, Switzerland) was used to generate amplified cDNA from total RNA of embryo samples. The same RNA samples used for RNA-seq were used (0.5 ng total RNA). Subsequently, the mRNA expression of the selected genes was measured in the amplified cDNA by qPCR on a Light Cycler 96 (Roche Diagnostics (Schweiz) AG, Rotkreuz, Switzerland) with the KAPA HiFi HotStart PCR Kit (Roche Diagnostics (Schweiz) AG) adding EvaGreen® Dye, 20 × in water (Biotium). The qPCR was performed in a reaction volume of 20 μL, consisting of 4 μL 5 × Kapa HiFi Buffer, 0.6 μL Kapa dNTP mix (10 mmol/L), 0.4 μL Kapa HiFi HotStart DNA polymerase, 0.6 μL of each primer (10 μmol/L), 1 μL Eva Green Dye, 11.8 μL water and 1 μL cDNA template. Cycle parameters of the PCR were 95 °C for 3 min, followed by 45 cycles of 98 °C for 20 s, specific annealing temperature for 15 s and 72 °C for 15 s, and then a melting step (95 °C for 10 s, 65 °C for 60 s and 97 °C for 1 s). Melting curves of the amplified PCR products were obtained for confirmation of specific amplification. A no-template control (RNA sample) was included for each primer pair. The Cq values (quantification cycle) determined for the selected genes were normalized against the geometric mean of two reference genes glyceraldehyde-3-phosphate dehydrogenase (*GAPDH*) and 18S rRNA (*RNS18*). Relative expression differences between VIT vs. IVC, VIT vs. Co and VIT vs. CO were calculated, and a *t*-test was performed in Microsoft Excel. *P*-values < 0.05 were considered significant.

**Table 1:** Primer sequences for genes used in gene expression analysis by quantitative real–time (qPCR)

Gene	Primer sequence 5′ to 3′	Product length, bp	Annealing Temp, C°	Accession No.	Ssc Gene ID
*EMP1*	F: TATACGGCGGTGAAGATGCC	130	63	NM_001099940	100,101,477
	R: AGGAAGAAGCGGTTGCCTTT				
*ANXA8*	F: ACCTCCACAGCTACTTTGCC	191	61	NM_001243599.1	100,155,930
	R: CTTGTAGTCGCCACTGGTGT				
*SLC10A1*	F: CTTTCACCGGCTTCCTGCTA	154	60	XM_001927695.5	100,153,302
	R: GGTCCAATGACTTCAGGGGG				
*PLET1*	F: ACACCGTCGAGCTACAAGTC	172	60	NM_213744.1	396,570
	R: TGTGTGGTGTGGGTTGTGAT				
*BTG2*	F: TTTCCTCTCCAGCCTCCTCA	142	61	NM_001097505.2	100,048,932
	R: GTAGCCAGAGCCCTTGGATG				
*SERPINE1*	F: AACCAGGCGGACTTCTCAAG	136	60	NM_213910.1	396,945
	R: TGCGGGCTGAGACGATAATG				
*GSTA4*	F: GTCTGCCTTTCCTCACCTCC	84	61	NM_001243379.1	100,152,951
	R: TTGCTGCCAGGTTCAAGGAA				
*SPP1*	F: GAGGGCAGCACTGACAGCCG	134	60	NM_214023.1	397,087
	R: GAAGGGCAGAGGCGAAGCCC				
*GAPDH*	F: AACTGCTTGGCACCCCTGGC	150	60	AF017079.1	396,823
	R: CTGGAGAGCCCCTCGGCCAT				
*RN18S*	F: GGGAGGAGGCTGACCGGGTT	84	60	NR_046261.1	100,861,538
	R: ATACATGCCGACGGGCGCTG				

## Results

### In vitro embryo survival, pregnancy, and farrowing rates of vitrified/warmed embryos

A total of 23 gilts were used as embryo donors and 394 embryos were recovered at blastocyst stage, from which 373 were used in the present study. In a first preliminary experiment, to evaluate the in vitro survival rate of embryos subjected to the vitrification protocol, 80 embryos were vitrified/warmed and cultured in vitro. The survival rate was 75% (60/80) and with a hatching rate of 63.3% (38/60). Subsequently, in vivo survival rate, pregnancy rates and farrowing rates of the vitrified/warmed embryos were also examined. For this purpose, 167 embryos were used, from which 150 embryos were transferred to recipient gilts (*n =*5) (30 embryos/recipient) and 17 were used to evaluate the in vitro survival and hatching rates which were 70.6% (12/17) and 16.7% (2/12). The pregnancy rates observed were 60% (3/5) on d 30 and 40% (2/5) on d 45, with a final farrowing rate of 20% (1/5) and 11 piglets born from one gilt.

For RNA-sequencing, a total of 126 embryos were collected and distributed to the VIT group (*n =* 56 embryos), IVC group (*n =* 35 embryos) and CO group (*n =* 35 embryos). In the VIT group, the in vitro survival rate of vitrified/warmed embryos followed by culture in vitro for 24 h was 82.1% (46/56) with a hatching rate of 15.2% (7/46). In the IVC group, the in vitro survival rate was 100% (9/9) with a hatching rate of 66.7% (6/9). Overall, the in vitro survival rate of vitrified/warmed followed by 24 h of in vitro culture was 77.1% (ranging 70–82%) with a hatching rate of 39.8% (15–63%).

### Impact of VIT and IVC procedures on the embryonic transcriptome

#### Exploring the alterations caused by VIT and IVC

**Fig. 1:**
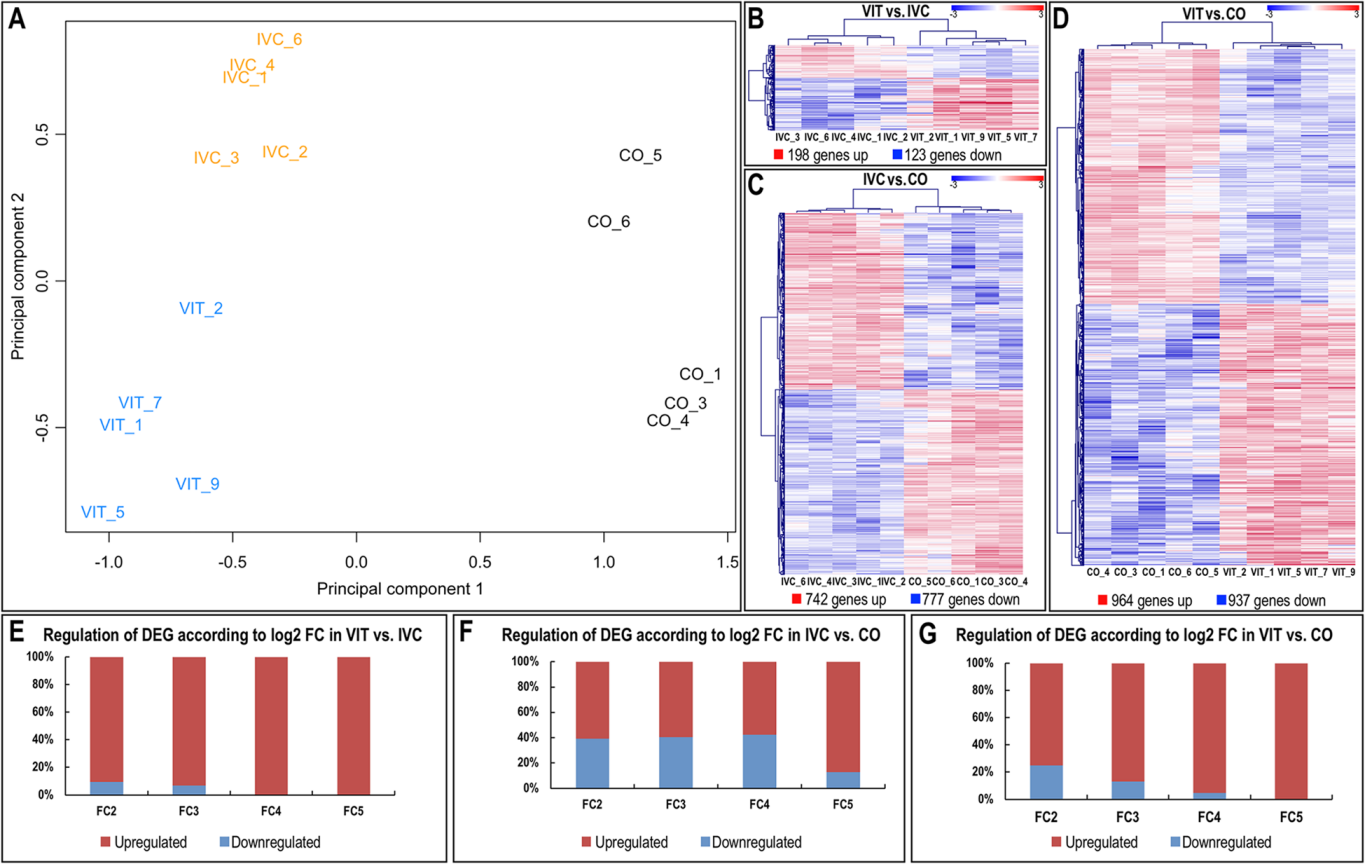
Comparative transcriptome analysis of embryos subjected to different technologies and controls (no treatment). **A** Principal Component analysis (PCA) based on the 500 genes with the highest variation of expression levels in the data set (vitrified embryos: VIT in blue; in vitro culture embryos: IVC in orange; and in vivo fresh control embryos: CO in black**). B–D** Dendrograms representing results of unsupervised hierarchical clustering (HCL) created with Pearson correlation coefficient by MeV. Rows show differentially expressed genes (DEGs) (cut off FDR 0.001) while columns represent embryo samples subjected to different technologies or controls **B**: VIT vs. IVC; **C**: IVC vs. CO and **D**: VIT vs. CO). Each sample represents a pool of embryos. Mean–centered expression values (log2 counts per million of sample–mean of log2 CPM of all samples) for the samples of the three embryo groups. Color scale is from − 3 (blue, lower than mean) to 3 (red, higher than mean). **E–G** Percentage of up– and downregulated genes according to different log2FC cut–offs in VIT vs. IVC embryos **E**, IVC vs. CO embryos **F**, and VIT vs. CO embryos **G**

RNA–seq analysis of VIT, IVC and CO embryos was performed, and transcripts derived from a total number of 9325 genes were identified in all embryos examined from the three different embryo groups (after filtering for a minimum number of read counts; Additional file 1–3: Table S1–3). Multidimensional scaling plots (principal component analysis, PCA) of normalized read count data revealed a clear separation of the samples of the three groups of embryos analysed (VIT, IVC and CO) (Fig. 1A). Then, in order to identify genes with altered gene expression in VIT vs. IVC as well as in VIT vs. CO and IVC vs. CO, statistical analysis for these three comparisons was performed. The total number of differentially expressed genes (DEGs) for these comparisons, based on the false discovery rate (FDR) < 0.001 and LR 13.80 set for the comparison IVC vs. CO, was 321 for VIT vs. IVC, 1901 for VIT vs. CO and 1519 for IVC vs. CO (Additional file 4: Table S4–6). Hierarchical cluster analysis of the DEG in Fig. 1 B–D clearly illustrates that a lower number of DEG was found for VIT vs. IVC comparison, compared to VIT vs. CO and IVC vs. CO. For VIT vs. IVC comparison, 198 genes were up–regulated and 123 were down–regulated in VIT embryos compared to IVC embryos (Fig. 1B). For IVC vs. CO comparison, 742 genes were up–regulated and 777 down–regulated in IVC compared to CO (Fig. 1C). For VIT vs. CO comparison, 964 genes were found up–regulated while 937 were down–regulated in VIT embryos compared to CO embryos (Fig. 1D).

To highlight genes with higher differences between embryo groups, a FDR of < 0.001 and log2 fold change (log2 FC) > 2 was applied. This analysis revealed 63 genes (6 downregulated and 57 upregulated); 253 (99 downregulated and 154 upregulated) and 369 (89 downregulated and 280 upregulated) for comparisons VIT vs. IVC, IVC vs. CO, and VIT vs. CO, respectively. A higher percentage of upregulated genes was found in VIT vs. IVC and VIT vs. CO than for IVC vs. CO after application of higher log2 FC cut–offs for all three comparisons (VIT vs. IVC, IVC vs. CO, and VIT vs. CO), as illustrated in Fig. 1E–G. For a log2 FC cut–off of 2, 90.5% (57 genes) of DEG were upregulated in VIT compared to IVC and all DEG were upregulated in VIT for log2 FC 4 and log2 FC 5 (17 and 11 genes, respectively) (Fig. 1E). Similarly, for log2 FC cut–off of 2, 75.8% of DEG (280 genes) were upregulated in VIT compared to CO. For log2 FC 4, the percentage of upregulated DEG further increased (93.2%, 41 genes), and all 8 DEG for log2 FC 5 were upregulated in VIT (Fig. 1F). By contrast, when IVC vs. CO was analysed, the regulation of DEG was more or less similar regarding the number of up– and downregulated DEG with 60% upregulated and 40% downregulated genes in IVC compared to CO, except when a log2 FC 5 cut–off was applied (Fig. 1F). The list of DEG upregulated and downregulated for each log2 FC cut–off can be found in Additional file 5: Table S7–9).

**Fig. 2:**
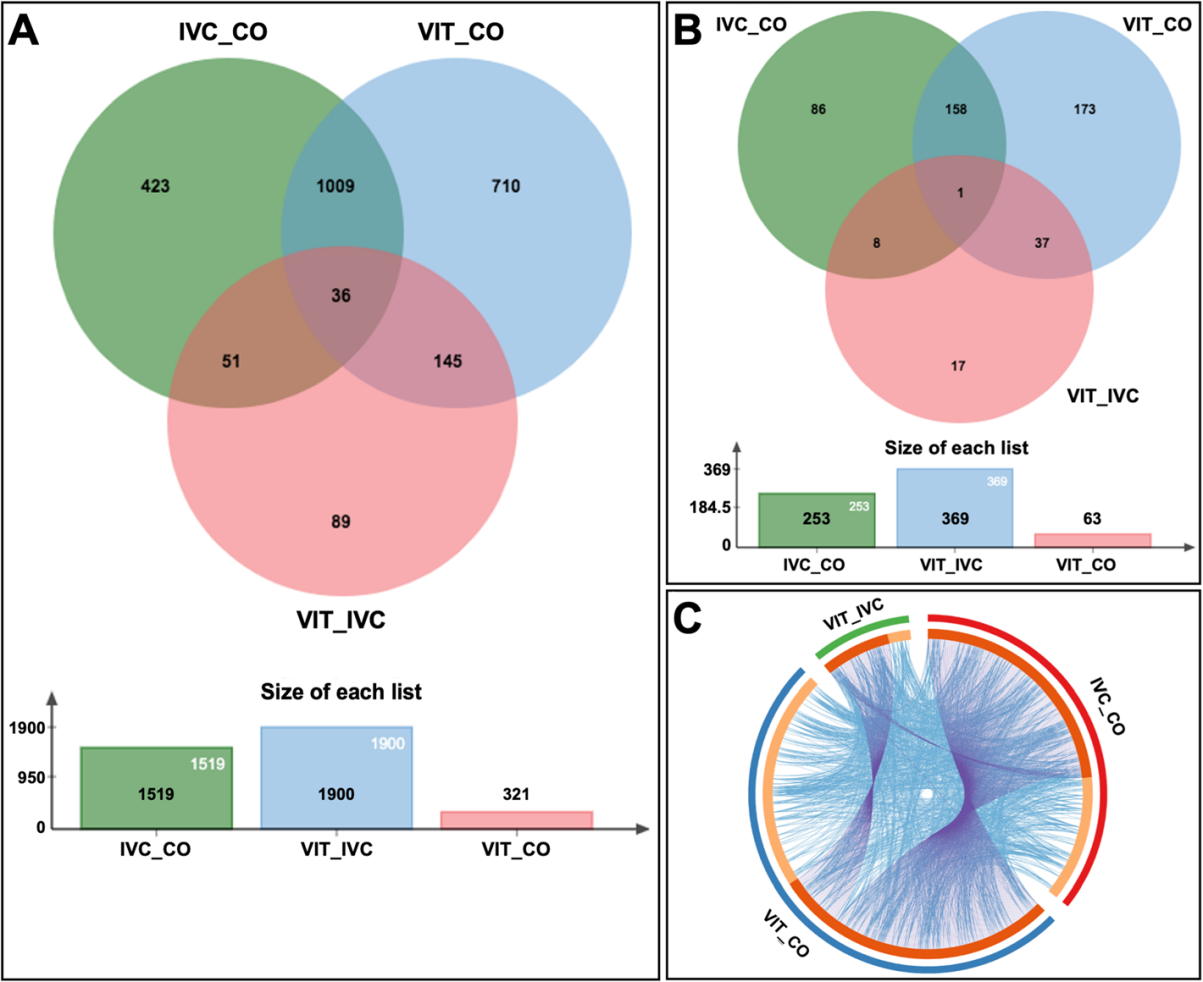
Overlap of differential expressed genes (DEGs) identified in the comparisons of embryo groups vitrification versus in vivo fresh control (VIT vs. CO); in vitro culture versus fresh control (IVC vs. CO); and vitrification versus in vitro culture (VIT vs. IVC). **A** Venn Diagram demonstrating overlaps of DEGs for comparisons among embryo groups with data analyzed with a cut–off FDR < 0.001. **B** Venn diagram representing DEG analyzed with a cut–off FDR < 0.001 and log2FC ≥ 2. **C** Circos plot representing DEG analyzed with a cut–off FDR < 0.001 and log2FC ≥ 2. The plot represents the overlap only at the gene level (purple lines) and at the shared functional term level (blue lines), i.e., link genes that belong to the same enriched ontology term. The circles represent the gene lists, genes contained in multiple lists in dark orange, and unique genes in light orange

The overlap of DEG among all comparisons is shown in a Venn diagram for DEG selected based only on FDR < 0.001 and based on FDR < 0.001 and log2 FC > 2 (Fig. 2 A–B, Additional file 6: Table S10). It is to be noticed an overlap of 1045 genes between VIT vs. CO and IVC vs. CO comparisons. Furthermore, the DEG shared among embryo group comparisons were illustrated with a circus plot for DEG based on a FDR of < 0.001 and log2 FC > 2 (Fig. 2C). These circos plots illustrate the overlap at the gene level by purple lines linking identical changes and at the shared functional term level by blue lines linking genes that belong to the same enriched ontology term. Besides, genes that hit multiple list comparisons are represented in different colors.

#### Functional analysis revealed potential regulatory biological functions and pathways affected differently by VIT and IVC procedures

**Fig. 3:**
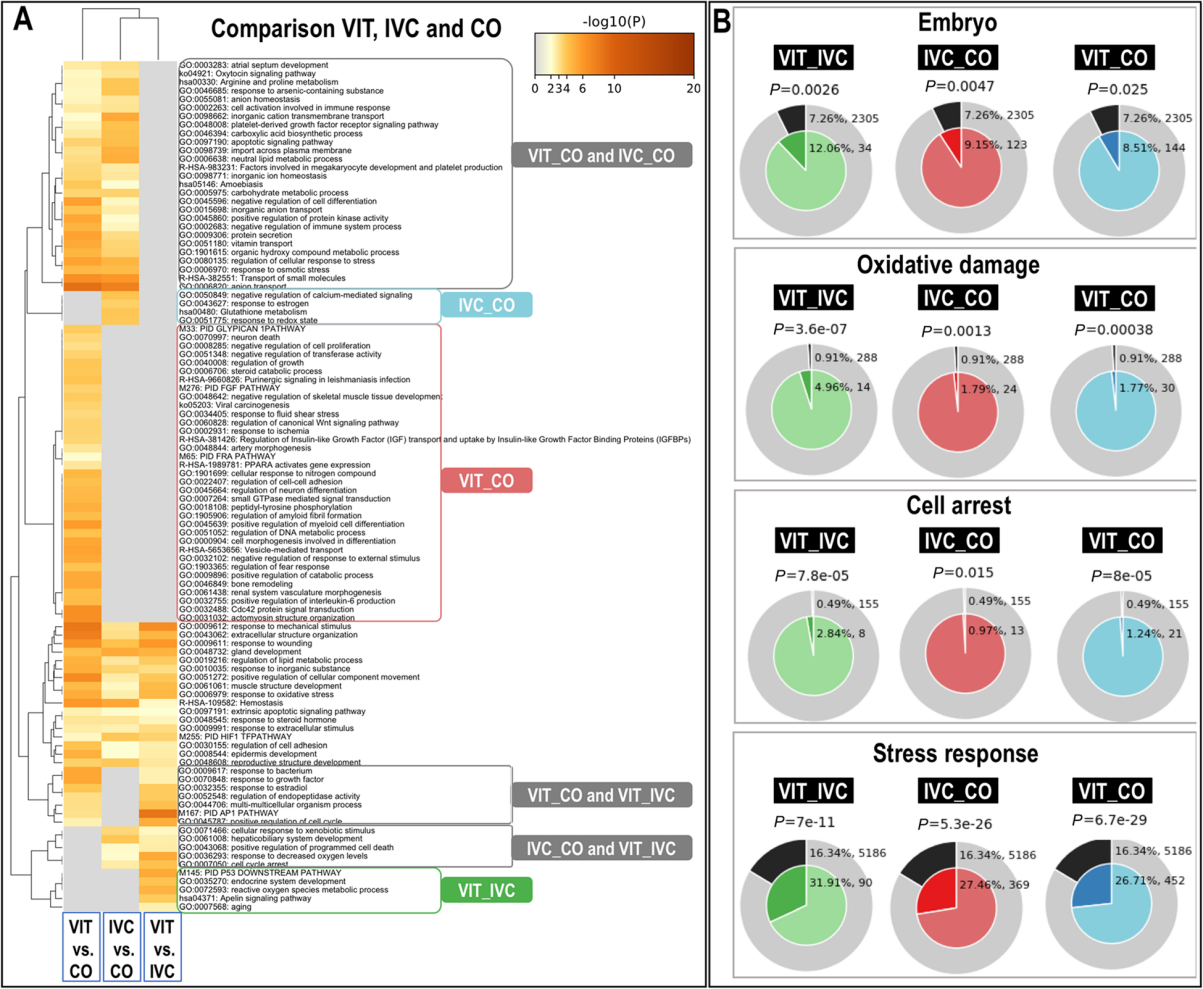
Functional enrichment analysis of differentially expressed genes (DEGs) identified in the comparisons of embryo groups: vitrification versus in vitro culture (VIT vs. IVC), in vitro culture versus control (IVC vs. CO) and vitrification versus in vivo control (VIT vs. CO), and using Metascape tool. **A** Heatmaps representing visualization of top 100 clusters of enriched terms across DEG lists with selected DEG (cut–off FDR < 0.001 and log2FC ≥ 2. In all plots, bar graph of enriched terms across DEG colored by *P*-values representing enriched clusters for score of ≥2**. B** Functional membership analysis for differentially expressed genes (DEG) with cut off FDR ≤ 0.001 identified in the comparisons of embryo groups vitrification versus in vivo control (VIT vs. CO); in vitro culture versus control (IVC vs. CO); and vitrification versus in vitro culture (VIT vs. IVC) using Metascape tool. Enrichment of DEG matching membership terms: “embryo”, “ oxidative damage”, “cell arrest” and “stress response”. The outer pie shows the number and the percentage of DEG in the background that are associated with the membership (in black); the inner pies show the number and the percentage of DEG in the individual input gene list that are associated with the membership. The *P*-values on the top of the pie charts indicates whether the membership is statistically significantly enriched in the list

To characterize the alterations of the embryo transcriptome by VIT and IVC procedures, gene ontology (GO) terms and pathways overrepresented for DEGs from the three different comparisons (VIT vs. IVC, VIT vs. CO, and IVC vs. CO) were analysed using functional annotation databases in Metascape tool (Additional file 7: Table S1–2). The analysis of GO terms was performed across the three DEG lists using a more strict DEG cut–off FDR 0.001 and log2 FC 2. The Fig. 3A illustrated the top 100 clusters of GO term and highlighted similarities and differences among lists in a heatmap. The GO terms specifically associated to VIT vs. IVC (Fig 3A, in green), such as reactive oxygen species metabolic process, endocrine system development, Apelin signaling pathway and aging. Genes altered in IVC vs. CO(Fig 3A, in blue) were related to response to redox state, glutathione metabolism, response to estrogen and regulation of calcium–mediated signaling. Genes altered in VIT vs. CO (Fig 3A, in red) were associated to regulation of canonical Wnt signaling pathway, regulation of growth and regulation of cell–cell adhesion, cell morphogenesis involved in cell differentiation, regulation of DNA metabolic process and vesicle–mediated transport. Moreover, genes altered in VIT vs. CO were associated with positive regulation of interleukin–6 production and Cdc42 protein signal transduction.

Additionally, other GO terms were found shared among two comparisons, with a higher number of terms associated to VIT vs. CO and IVC vs. CO and with a much lower number of GO terms associated to VIT vs. IVC and VIT vs. CO (response to estradiol, AP1 pathway, positive regulation of cell cycle) or to VIT vs. IVC and IVC vs. CO (positive regulation of programmed cell death, cell cycle arrest, response to decrease oxygen levels) (Fig. 3A). A group of GO terms were also identified for the three comparisons (VIT vs. IVC, IVC vs. CO and VIT vs. CO). For example, response to mechanical stimulus, extracellular structure organization, response to wounding and response to oxidative stress were highly enriched in VIT vs. IVC and VIT vs. CO when compared to IVC vs. CO.

In search of specific alterations that could be induced by the VIT procedure on the embryos, Metascape Membership tool was used to obtain enrichment of DEG matching key terms such as: “embryo”, “cell arrest”,” oxidative damage” and “stress response”. Figure 3B shows the results of this analysis for the three DEG list comparisons VIT_IVC, VIT_CO, and IVC_CO. The assigned DEG to each membership and the specific functional terms can be found in Additional file 7: Tables S3–10. The outer ring of each pie (grey) shows the number and the percentage of genes in the background that are associated with the membership term(s) (in black). While the inner of each pie shows the number and the percentage of genes in the individual input gene list that are associated with the membership term. The *P*-value at the top of each pie indicates whether the membership term is significantly enriched in the list. For the membership term analysis “embryo”, the analysis showed that 12.06%, 9.15% and 8.51% DEG were related to this GO term from the VIT_IVC, VIT_CO and IVC_CO lists (*P*-values: 0.0026, 0.025 and 0.0047), respectively. For the rest of the membership terms analysed “cell arrest”, “oxidative damage” and “stress response”, significant values were also found in the three comparison lists in Fig. 3B.

#### Potential canonical pathways, biofunctions, and upstream regulators affecting embryo development in embryos subjected to VIT and IVC

**Fig. 4:**
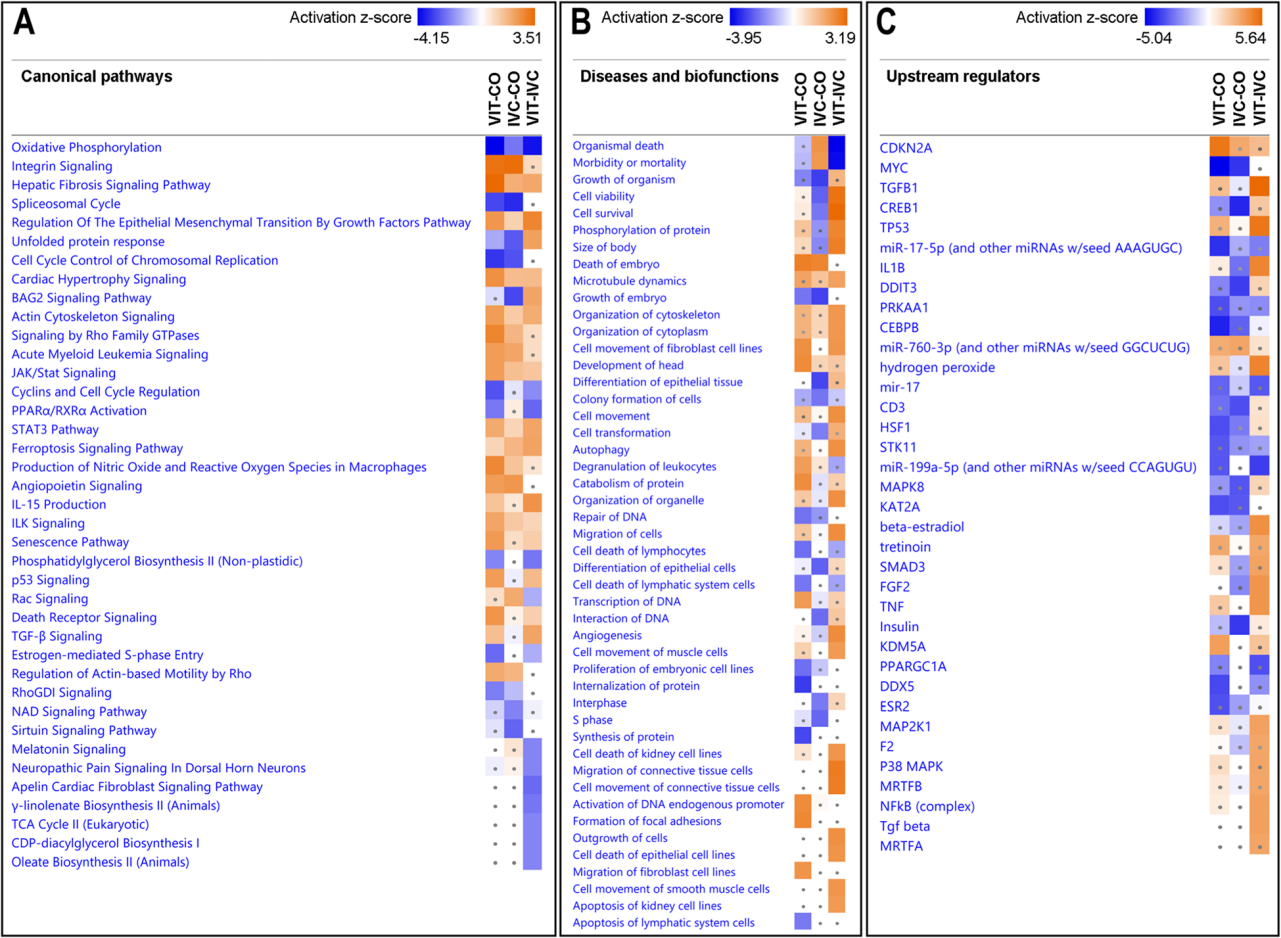
Heatmaps illustrating selected canonical pathways **A**, diseases and biofunctions **B**, and upstream regulators **C** identified across the three DEG lists: vitrification versus control (VIT–CO), in vitro culture versus control (IVC–CO) and vitrification versus in vitro culture (VIT–IVC), using Ingenuity Pathway Analysis software core analysis and Comparison Analysis tool

Ingenuity Pathway Analysis software core analysis was performed for the VIT vs. IVC as well as for VIT vs. CO and IVC vs. CO datasets and results were compared using Comparison Analysis. The IPA regulation Z-score was used to identify activated and inhibited significantly enriched (*P*-value < 0.01) canonical pathways, diseases and biofunctions, and upstream regulators. Selected canonical pathways, biofunctions, and upstream regulators are illustrated in Fig. 4A, B and C, respectively. Based on these results, for the VIT vs. IVC comparison, significant inhibited enriched canonical pathways were identified: oxydative phosphorylation, cyclins and cell cycle regulations and similar to VIT vs. CO comparison. Also, Phosphatidilglycerol Biosintheysis II, γ–linolenate biosynthesis II (animals), CDP–diacylglycerol biosynthesis I, oleate biosynthesis II (animals and fungi) were also inhibited enriched canonical pathways. By contrast, enriched canonical pathways in VIT vs. IVC as significantly activated (Z-score > 2) were related to regulation of the epithelial mesenchymal transition by growth factors pathway, BAG2 signalling pathway, IL-15 production and TGF-β signalling.

For the VIT vs. CO comparison, other enriched canonical pathways (Fig. 4A) were identified as significantly activated (Z-score > 2) related to Integrin Signalling, Actin Cytoskeleton Signalling, Senescence Pathway, p53 signalling, and also TGF–β signalling. By contrast, oxidative phosphorylation, cell cycle control of chromosome replication, cyclins, and cell cycle regulation were significantly inhibited in VIT_CO. For the IVC_CO comparison, enriched canonical pathways significantly activated (Z-score > 2) were also involved in Integrin signalling. While pathways significantly inhibited (negative Z-score > 2) were BAG2 signalling pathway and Sirtuin Signalling Pathways.

**Fig. 5:**
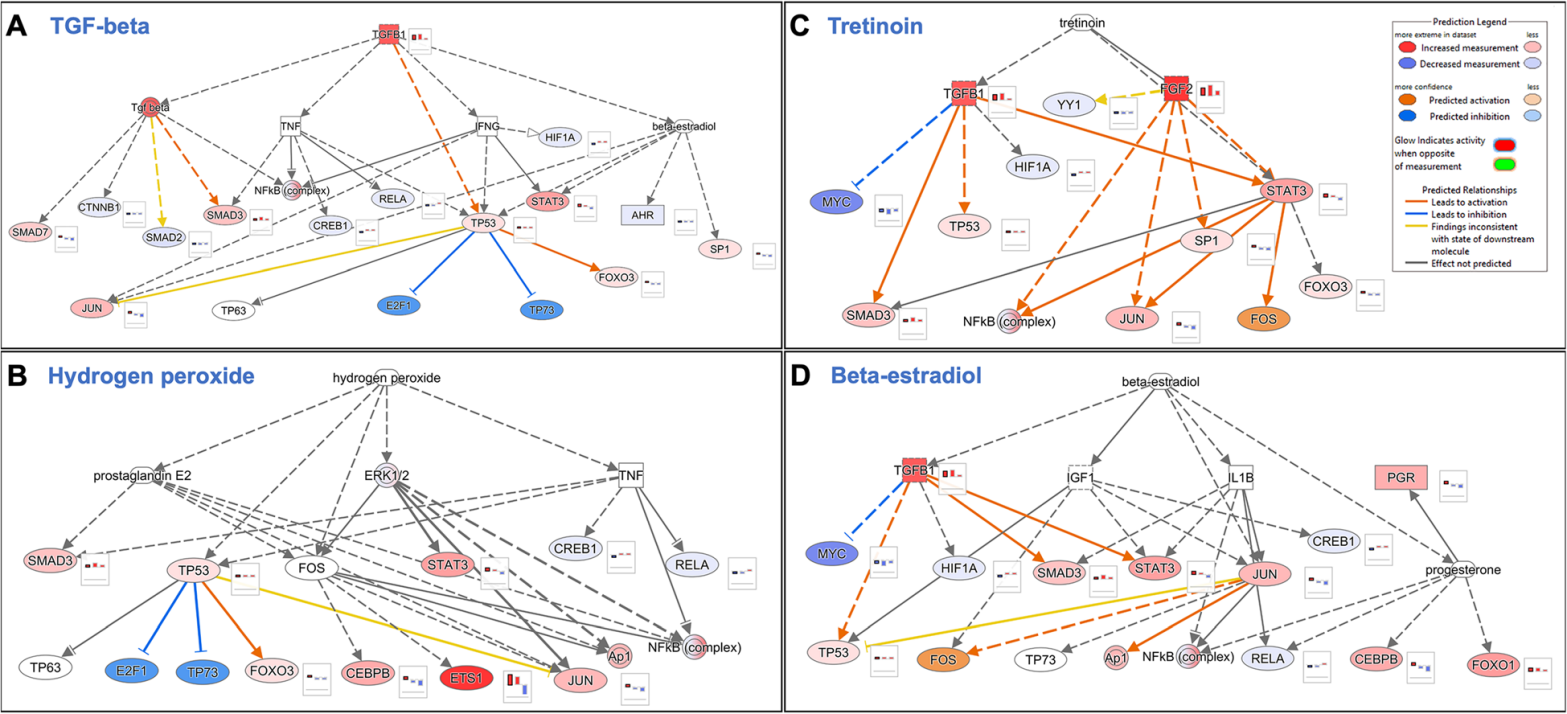
Networks illustrating potential upstream regulators identified for the comparison vitrification versus control (VIT vs. CO), using Ingenuity Pathway Analysis software core analysis: **A** The network of TGFB; **B** The network of Tretinoin; **C** The network of Hydrogen peroxide and **D** The network of Beta–estradiol. The barplot next to the genes shows regulation for VIT vs. IVC, VIT vs. CO, IVC vs. CO.

Additionally, diseases and biofunctions analysis (Fig. 4B) based on the same Z-score was performed for the three DEG lists (VIT vs. CO, IVC vs. CO and VIT vs. IVC) (filtering all cancer terms). In VIT vs. IVC, cell viability and cell survival were significantly activated, while they were inhibited for IVC vs. CO. Furthermore, organization of cytoskeleton and organization of organelles as well as phosphorylation of proteins were also active in VIT vs. IVC. By contrast, the biofunction death of embryo was highly activated for VIT vs. CO and IVC vs. CO comparisons, while growth of the embryo was inhibited. Differentiation of epithelial cells and tissues was found only inhibited in IVC vs. CO compared to the other comparisons. While for the comparison VIT vs. CO, internalization of proteins and Synthesis of proteins and Repair of DNA were the functions significantly inhibited. Finally, upstream regulator analysis results were compared (Fig. 4C), including transcription factors, cytokines, small non–coding RNAs, receptors, kinases, chemical molecules, and pharmacological agents that can potentially regulate gene expression. We focused on upstream regulators, which are genes identified in our study or in chemical components that could have an effect in the embryo in vivo or in vitro during IVC. The top activated and inhibited up stream activators based on a z–score 2 are represented in Fig. 4C (*P*–value 0.01; Z–score 3.3). For the VIT vs. IVC comparison, TGFB1 (Fig. 5A), TP53, IL1B, FGF2 as well as hydrogen peroxide (Fig. 5B) and beta–estradiol (Fig. 5D) were pointed as possible activators. Interestingly, Tretinoin, the active form of Vitamin A or retinoic acid, although had a z–core below 2, was also found activated in VIT vs. IVC as well as in VIT vs. CO comparison when compared to IVC vs. CO (Fig. 5C). In VIT vs. CO, CDKN2A is the top activator, while MYC and CEBPB were among the top inhibitors. In IVC vs. CO, MYC was also among the top inhibitors together with insulin.

#### Unveiling the specific impact of the vitrification process

**Fig. 6:**
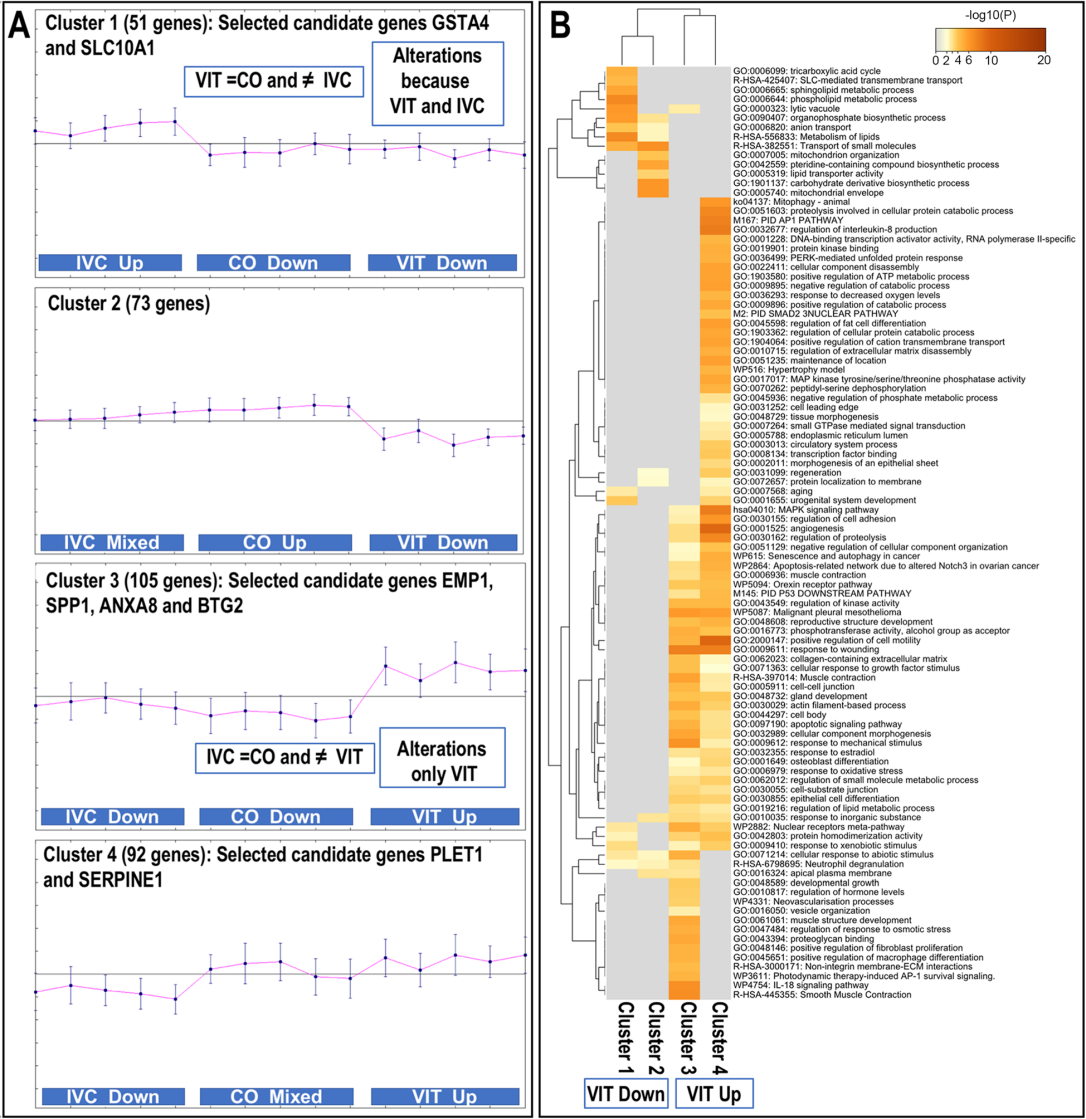
Cluster analysis of differentially expressed genes (DEG) identified in vitrified embryos compared to in vitro culture (VIT_IVC) and associated GO biological functions and pathways. **A** Cluster analysis of 321 differential expressed genes (DEG) identified in VIT_IVC comparison pointing to genes altered due to VIT and their similarities to CO or IVC embryos by using MeV. **B** Heatmaps representing visualization of top 100 clusters across DEG lists in clusters 1 to 4, showing GO biological functions and pathways associated to down and upregulated genes in VIT compared to IVC

For this purpose, we focused on the comparison VIT vs. IVC and performed clustering analysis of the 321 DEG identified in VIT vs. IVC by using MeV tool and Pearson correlation. This analysis provided 4 different clusters of expression profiles obtained for the VIT vs. IVC DEGs (Fig. 6A) and highlighted similarities and differences of the gene expression profile of the 321 DEG across all samples. Clusters 1 and 4 showed genes with similar expression in VIT and CO but different in IVC, while clusters 2 and 3 illustrate genes with similar expression in IVC and CO but different in VIT. In addition, clustering results showed that genes in cluster 2 (73 genes) were downregulated in VIT while cluster 3 (105 genes) were upregulated in VIT compared to IVC and CO (Fig. 6A).

Subsequently, the biological functions associated to the genes included in each cluster were analysed in Metascape and results are illustrated in the heatmap of Fig. 6B. Genes in cluster 1 (downregulated in VIT compared to IVC) were related to GO terms such as SLC-mediated transmembrane transport, sphingolipid metabolic process, phospholipid metabolic process and metabolism of lipids and aging. Genes in cluster 2 (also downregulated in VIT compared to IVC) were related to GO terms associated to carbohydrate derivative biosynthesis process, transporter small molecules and lipid transporter activity and regeneration. Clusters 3 and 4 (upregulated in VIT compared to IVC) shared more GO terms in comparison to cluster 1 and 2 and were linked to MAPK signalling pathway, regulation of cell adhesion, angiogenesis, negative organization of cell component organization, senescence, reproductive structure development, apoptotic signalling pathway and response to wounding. Specifically, in cluster 3, genes were related to developmental growth, regulation of hormonal levels and regulation of response to osmotic stress, while in cluster 4 were associated to aging and regeneration, tissue morphogenesis, response to decreased oxygen levels, regulation of interleukin-8 production and mitophagy-animal (degradation mitochondria).

#### Selecting candidate genes as markers of transcriptional signature of embryonic VIT-damage and/or VIT-healing processes

Genes showing high differences in gene expression between VIT vs IVC (base on log2 FC) and also with marked different expressions profiles across VIT, IVC, and CO, derived from the clustering analysis (Fig. 6), were selected as markers of transcriptional signature of embryonic VIT-damage or healing processes. From cluster 1, *GSTA4* and *SLC10A1*, which were downregulated in VIT (and also CO) compared to IVC were selected. From cluster 3, selected genes were *EMP1*, *ANXA8*, *SPP1* and *BTG2*, which were upregulated in VIT embryos compared to IVC (and also CO) embryos. From cluster 4, selected genes were *PLET1* and *SERPINE1*, which were upregulated in VIT (similar to CO embryos) but contrary to IVC (upregulated). The biological functions linked to these 8 candidate genes were further investigated and is illustrated in Additional file 7: Table S8 and also for each individual genes. Interestingly, *PLET1, SERPINE1, SPP1* and *ANXA8* were associated to wound healing functions and *SERPINE1, SPP1* together with *BTG2, GSTA4* and *SLC10A1* were related to response to different stimulus, as shown in Additional file 7: Table S8.

#### Validation of embryonic gene expression results by qPCR of selected candidate genes

**Fig. 7:**
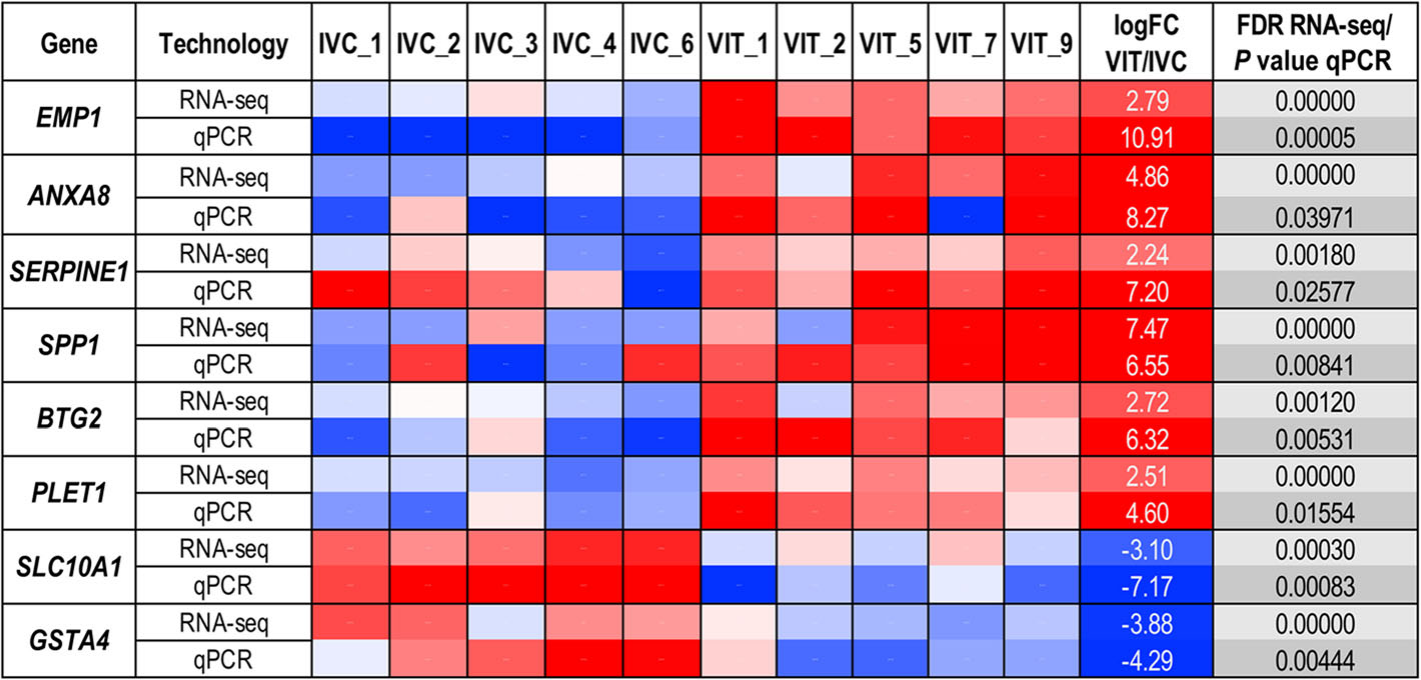
Confirmation of RNA sequencing results by qPCR: Heatmap of qPCR and RNA-seq data for eight selected genes. To illustrate correlation of RNA seq and qPCR data relative expression levels (mean-centered log2 expression values) are shown as a heatmap. Red color means higher and blue lower expression levels of the gene compared to the mean of all samples, respectively (from 2 over 0 to −2). Statistical differences are represented by FDR for the RNA-seq data and by *P* value for qPCR data (*P* < 0.05)

Gene expression results of the 8 selected genes for VIT vs. IVC embryos performed by qPCR was illustrated in Fig. 7. Differences in embryonic gene expression between VIT embryos and IVC embryos observed by RNA-seq results were confirmed for all of 8 genes by qPCR (*P* value < 0.05). Again, *SLC10A1* and *GSTA4* embryonic gene expressions were downregulated (Fig. 7, in blue) while *EMP1*, *ANXA8*, *SPP1*, *BTG2*, *SERPINE1* and *PLET1* were upregulated (Fig. 7, in red) in VIT when compared to IVC embryos. Additionally, Additional file 7: Table S9 illustrated embryonic gene expression profiles for all genes and also reference genes across all samples (VIT, IVC and CO) obtained by qPCR.

## Discussion

Our study provided the transcriptional signature of porcine embryos that survived successfully the VIT procedure by RNA-sequencing, revealing important alterations that might reflect the molecular cryo-damage but also the molecular healing process in the embryos to overcome the impact of VIT. To unveil this, the study focused mainly on the alterations of the VIT procedure on the embryonic gene expression by comparing VIT vs. IVC embryos. Additional comparisons of both VIT and IVC embryos to CO embryos were performed merely as a confirmation of the alterations of the VIT procedure, since VIT and IVC procedures share both 24 h of embryo culture. The study design and the embryonic functional alterations identified associated to VIT procedure are discussed below.

### Strategies to study the specific impact of VIT on embryonic gene expression and their limitations

We hypothesized that embryonic gene expression alterations necessary to overcome the vitrification damages and allowing embryo recovery are reflected in the embryos that successfully survived the VIT procedure and they could be used to evaluate the efficiency of the vitrification procedure. Mostly, the evaluation of the success of vitrification protocols is based on in vitro survival rates, which implies that an in vitro culture step is performed to evaluate the restructuration of the embryo and its quality after vitrification/warming [39–41]. This makes the analysis of specific effects of VIT on embryos very difficult and involves important limitations: 1) If the VIT embryos are used for RNA–seq analysis directly after warming, these embryos may include an unknown proportion of embryos which would not recover (did not survive VIT) and recovering ones, affecting the gene expression analysis; 2) If the VIT is followed by 24 h IVC to allow the embryos to recover, the challenge is to distinguish VIT from IVC effects.

It is known that IVC induces a strong delay in embryo development [55, 56]. Furthermore, the IVC effect on VIT and IVC embryos might be slightly different since VIT embryos need to recover from VIT procedure and re-expand while the IVC embryos just adapt to IVC conditions and continue their development. Altogether, it is difficult to find an appropriate control that allows to separate these two components.

Therefore, our strategy focused on the VIT vs. IVC comparison to reveal specific VIT alterations, including the 24 h IVC effect present in both groups. Additional comparisons of VIT vs. CO and IVC vs. CO were performed to see if the observed overall transcriptome were conclusive and to further support the reliability of the alterations of the VIT procedure itself, since these two comparisons might also reflect the sum of alterations associated to the effect of the embryo development and the short in vitro culture. Particularly, the VIT vs. CO comparison allowed to evaluate the global gene expression changes induced by vitrification and in vitro embryo recovery and embryo development. The IVC vs. CO comparison allowed to have an overview of gene expression changes induced by in vitro culture itself and also by the embryo development progression. The PCA and differential gene expression results clearly showed that there is a strong effect of both IVC and VIT on the embryonic gene expression when compared to CO. The comparison of datasets revealed the IVC as the main factor affecting embryonic gene expression and a much lower number of DEG but with high expression differences for many of the DEG for VIT vs. IVC comparison, which is representing the specific VIT alterations. Moreover, the large overlap in gene alterations between VIT vs. CO and IVC vs. CO confirmed the consistency of the findings.

### The effects of VIT on the embryonic transcriptome

#### Comparing the genes altered in VIT embryos to other studies

From the total number of DEG identified in VIT vs. IVC more DEG (60%) showed higher expression in VIT compared to VIT with increasing percentage for DEG with log2 FC > 2. Recently, Cuello et al. [39] also analyzed the gene expression of blastocysts subjected to VIT procedure by microarray and detected 182 DEG (un-adjusted *P*-value < 0.05) in VIT compared to CO embryos. Although they stated 205 DEG, the list contained only 182 DEG due to redundant gene IDs (in some cases more than one probe set per gene on the array). They also observed an increase in the number of upregulated DEG in VIT when a log2 FC cut–off of 2 or higher was applied. A comparison to the results of the study by Cuello et al. [39] revealed an overlap of only 20 DEG. The comparison to the 1901 DEG from VIT vs. CO resulted in 49 DEGs in common to both studies (Additional file 6: Table S11). A final comparison was performed with all genes detected as considerably expressed in our study (9324) and the 182 DEG of Cuello et al. [39] (since a list of all detectable genes was not published by the authors) and found 129 DEG in common. These differences between studies might be due to the different techniques used to analyze gene expression, microarrays versus RNA–seq, and different data processing and statistical analysis. In Cuello et al. [39], no information was provided about filtering for present (detectable) probe sets and no adjustment for multiple testing has been performed. In addition, differences could be also associated to the different breeds used. Montagner et al. [36] reported different in vitro survival rates between porcine breeds [36]. Furthermore, the low overlap could be related to differences in in vitro survival rates between our study (82% for VIT vs. 100% for CO) and Cuello et al. [39] (VIT: 96.1% vs. CO: 100%). It could be possible that in Cuello et al. [39] the vitrification protocol is perfectly optimized leading to only very minor effects on gene expression due to VIT that can be detected 24 h later. However, the in vitro survival rates of vitrified/warmed embryos obtained in our study (70–82%) were in the same range to other studies using the same vitrification protocol [14, 57], making the results interesting for the use as biomarkers for VIT effects.

Further comparisons of our study with other studies in the literature performing extensive embryonic gene expression analysis by RNA-seq to find common alterations of the VIT procedure was complicated since in some cases vitrification was performed using other protocols or devices [40], embryos were subjected to other biotechnologies before the vitrification step [40] or VIT was performed in other species and at different developmental stages [58]. For example, a higher number of upregulated genes (782) was also found in bovine elongating conceptuses (d 14), with only 145 downregulated in VIT compared to CO embryos (of 927 total DEG identified) [58]. These embryos were vitrified on d 7, transferred to heifers and collected on d 14 of development, and further analyzed by RNA–seq [58]. Zhang et al. [40] analyzed gene expression of embryos obtained after somatic cell nuclear transfer (SCNT), vitrified and used for gene expression analysis after a short IVC of 6 h (Additional file 6: Table S11). The paper of Zhang et al. [40] contains different statements about significant differences in expression levels between SCNT–VIT (vitrification group) vs. SCNT–CNT (control group). It seems they found 540 DEG (FDR 5%) between the VIT and the control group, but only showed 178 DEG in their supplemental material. However, when results of Zhang et al. [40], Cuello et al. [39], and our study were compared, no overlap was found between all the three studies. Seven DEG were in common for Cuello et al. [39] and Zhang et al. [40] (*ART3*, *CELA2A*, *DUSP6*, *NQO1*, *NTRK3*, *PGM5*, *PHLDA3*) and 8 genes between ours and Zhang et al. (*ACTA1*, *BHLHE40*, *CYP1A1*, *EMP1*, *F2R*, *PIP4K2B*, *SLC10A1*, *UCHL1*) (the complete list of comparisons among studies can be found in Additional file 6: Table S11). This raised the question how researchers can depict and associate specific gene alterations to VIT in search of embryo markers, if despite each study showed that the vitrification procedure alters the gene expression of porcine blastocysts, the overlap of altered genes among studies was very small. This also highlights the importance of reporting all details of the embryo protocols, gene expression analysis, and gene annotation. Despite of the small overlap at the gene level, at least common biological functions and pathways were identified related to VIT among studies.

#### Vitrification induces common functional perturbations across studies

In agreement with Cuello et al. [39], our study showed that the main biological functions and pathways were associated to transforming growth factor beta (TGF beta) pathway, p53 signaling pathways, and cellular senescence. The identified upregulation of genes involved in cell cycle pathway (*CDKN1A*) and signaling pathways regulating pluripotency of stem cells (*BMPR1B*) are representing key pathways in embryo development. Besides, an increase in *CDKN1A* induced by *TP53INP* in both studies, has been associated to conditions of reparable damage or transient stress [59].

Searching for molecular insights that could explain the lower pregnancy rates obtained with vitrified embryos when compared to fresh, we observed that different members of the TGF–beta pathway were upregulated in VIT embryos (VIT vs. IVC: *TGFB1* and VIT vs. CO: *TGFB1, TGFBR1*, *TGFBR3*), which are associated with tissue remodeling events and reproductive processes, and particularly in embryo implantation process [60]. Cuello et al. [39] also found upregulation of *TGFB1* gene expression in VIT embryos. TGFB secreted by the murine blastocyst induces apoptosis of uterine epithelial cells, and in this way, playing a key role in the embryonic signaling to the endometrium during implantation [61].

Genes associated to different members of the heat shock protein (HSP) family were also detected as altered in VIT vs. IVC, as well as in VIT vs. CO and IVC vs. CO comparisons. Thus, it should be considered that VIT but also IVC affects HSP gene expression. Messenger RNAs for *HSPA5* and *HSP70.2* were upregulated in VIT embryos compared to IVC and also in VIT vs. CO. The expression of specific HSPs in embryos and also oocytes after vitrification has been reported [16, 38, 62]. Particularly, *HSPB1* and *HSPA1A* were also found as upregulated in vitrified blastocyst compared to controls [38, 39]. The cell increases HSP synthesis in response to heat stress, to other environmental stress or to reactive oxygen species, in order to protect against apoptosis induced by these stimuli [63, 64]. By contrast, other HSPs were found as downregulated in VIT vs. CO (*HSPD1*, *HSPA8*, *HSPA90AA1*, *HSPE1*, *HSPH1*), which might point to specific alterations to overcome the VIT effect and different roles of specific HSPs. The up- and also down-regulation of HSP in embryos has been observed in other studies, implying that HSPs play vital roles during embryo development [65, 66]. Besides, the Y–box binding protein 3 (YBX3), a cold shock protein, involved in cellular hyperosmotic response, negative regulation of intrinsic apoptotic signaling pathway in response to osmotic stress, and in utero embryonic development, was also found as upregulated in VIT vs. IVC and also in VIT vs. CO. Focusing on both heat and cold stress–responsive gene expression alterations might help to provide a better understanding of the stress tolerance mechanisms in the embryos to overcome the damage induced by vitrification.

### Towards improving VIT-related embryonic alterations: biomarkers of VIT-damage and VIT-healing processes and clues for intervention

The embryonic gene expression alterations due to VIT procedure reported in our study might reflect the activation of genes as a part of the repair and protection processes and stress adaptation response of the embryo to successfully overcome the VIT process impact. Thus, the identification of specific genes among these datasets that could be used as potential biomarkers for embryo quality or targets for intervention represents a valuable tool to evaluate the efficiency of the VIT procedures. With this aim, we focused on 8 genes showing the greatest changes (based on log2FC) among the 321 DEGs in VIT vs. IVC and with GO terms associated to wound healing among others. Furthermore, these genes were further validated by qPCR to confirm the RNA-seq data and further support the potential use of these genes as biomarkers.

The gene *EMP1* (epithelial membrane protein 1), was found as upregulated in VIT vs. IVC and also in VIT vs. CO (log2 FC = 2.8 and 4.1 respectively; cluster 3). Similar results were reported for porcine SCNT vitrified embryos [40], rabbit vitrified embryos [67], and also slow frozen rabbit embryos [68] compared to control embryos. These alterations have been linked to fetus development failure [67]. EMP1 has key roles in cell proliferation, differentiation, cell adhesion and epithelial junction formation [69]. Moreover, it has been attributed a role in tissue repair and wound healing [70], which could be linked to the healing ability of the embryo to overcome the VIT cryo-damage.

Gene expression of secreted phosphoprotein 1 (*SPP1*) was found also upregulated in VIT vs. IVC in our study. By contrast, Gupta et al. [71] reported a downregulation of *SPP1* in bovine blastocyst subjected to slow freezing at the morula stage compared to control. SPP1 plays multiple key roles in implantation and placentation [72], decreasing polyspermy in porcine oocytes [73], and improving in vitro development of porcine embryos and decreasing apoptosis in embryos [74]. In porcine embryos, SPP1 has been proposed to decrease cell death by reducing reactive oxygen species and nitric oxide production by injured tissues [74]. In this sense, several studies support the key function of SPP1 in response to injury and its active role in healing processes [70, 75], suggesting this gene as a biomarker of the VIT-healing or recovery process of the embryo.

*SERPINE1* (also known as PAI-1), was also found upregulated in VIT vs. IVC. Similarly, higher expression of *SERPINE1* was observed in embryonic tissues derived from retarded embryos compared to normal embryos [76]. SERPINE1 appears to be a key regulator of tissue repair [77] and determinant of cellular senescence [78]. An increased in *SERPINE1* mRNA levels in our study might be associated to a certain loss of cellular power in division and growth. On the other side, studies proposed SERPINE1 as a marker to evaluate human umbilical cord-derived mesenchymal stem cells after cryopreservation and long-term culturing [79] and its use as supplement during bovine sperm cryopreservation to preserve sperm acrosome integrity [80]. These findings highlight the potential of SERPINE1 for intervention strategies to improve the impact of the VIT procedure on the embryo.

Higher gene expression of *PLET1*, which has a potential role in cell lineage specification and defining stem cell potency [81], was observed in VIT vs. IVC. Murray et al. showed that high PLET1 levels favors differentiation towards the trophoblast giant cell lineage, whereas lack of PLET1 preferentially induces syncytiotrophoblast formation [82]. Furthermore, PLET1 has been referred as a wound-healing marker and also as a marker for migratory cells, and as a key gene for tissue repair following damage of different tissues [83]. Overall, it makes PLET1 an interesting candidate for VIT recovery.

The gene expression of *SLC10A21* was found as downregulated in embryos that successfully survived the VIT process. Huang et al. reported that *SLC10A1* gene expression was strongly upregulated (FC = 12) in degenerative embryos compared to control blastocysts obtained from IVF [84]. SLC10A1 among other solute carriers was also highly reduced in placentas derived from SCNT embryos compared to in vitro produced embryos and associated to an inefficient trafficking of macromolecules, which can affect fetal development [85].

BTG2, which has been related to cell growth, differentiation, and survival. [86], was upregulated in VIT embryos compared to IVC. Indeed, the induction of this gene is usually considered to be associated with temporal cell cycle arrest. Although we did not find associations of this gene with alterations of embryo cryopreservation in the literature, Lorda-Diez et al. demonstrated BTG2 overexpression in limb mesodermal progenitors increases oxidative stress and induces cell death and cell senescence [87]. Sustained overexpression of BTG2 in human embryonic stem cell exposed to ionizing radiation has been suggested as a part of the “gene expression signature” [85], which suggests it could be also a good marker of the embryo VIT damage.

Gene expression of *ANXA8* (annexin A8) was also strongly increased in VIT vs. IVC (and also in VIT vs. CO) (log2 FC = 4.9 and 4.6, respectively). Similar results were obtained by Cuello et al. [39] in VIT porcine embryos and by Zhou et al. [88] in porcine parthenogenetic embryos, and also SCNT compared to in vivo embryos. ANXA8 is one of the least characterized members of the annexin (ANXs) family, which is a group of Ca^2+^–dependent phospholipid–binding proteins involved in many important biological processes including calcium signaling, cell growth, inflammation and apoptosis [89]. It has been reported that vitrification negatively impacts porcine embryo quality, augmenting levels of apoptosis [90]. Thus, we hypothesized that ANXA8 could activate apoptotic process in embryos. Although *ANXA8* is not a typical apoptosis gene, it showed higher deregulation in VIT embryos than CASP genes family (*CASP8* and *CASP10* also deregulated in VIT; log2 FC < 2) or than *BAX* and *BCL2* genes, for which no differences were found in VIT vs. IVC (or VIT vs. CO). Similarly, Castillo-Martín reported that *BAX* and *BCL2L1* mRNAs were not altered in pig embryos subjected to vitrification [38]. On the other hand, *ANXA8* has been particularly associated with late endosomes and is required for normal morphology and intracellular actin-based motility and distribution [91].

The gene *GSTA4* (glutathione S–transferase alpha 4) was found downregulated in VIT vs. IVC embryos (log2 FC = − 3.9). In fact, *GSTA4* is included in cluster 4 embryo gene expression profiles, containing genes only upregulated in IVC compared to VIT and also CO indicating that IVC is upregulating this gene but VIT is counteracting this upregulation leading to expression levels similar to control embryos. *GSTA4* gene expression was also increased as a result of heat shock in morula stage embryos together with other genes related to oxidative stress [92]. An increased *GSTA4* gene expression in embryonic stem cells has been attributed to potential defense mechanisms against reactive oxygen species [93]. Considering that these studies shared the IVC procedure, *GSTA4* perturbation could be associated with stress in the embryo and expression after VIT could be an indicator of embryo quality and potential for pregnancy success which needs further detailed studies for confirmation.

The VIT derived embryonic alterations reported here could also be used as new avenues for intervention strategies. For example: 1) by supplementation of the media before or during VIT or IVC procedures with natural or synthetic molecules downregulated in the embryos compared to controls; or 2) by adding synthetic inhibitors to block or avoid the negative effects of specific genes up-regulated in VIT or IVC that cause damage in embryo metabolism or developmental processes. These strategies could reduce the detrimental effect on embryo quality and consequently early pregnancy loss or later impacts in adulthood.

In this regard, our study showed that a set of other HSP genes was downregulated in VIT (*HSPD1*, *HSPA8*, *HSPA90AA1*, *HSPE1*, *HSPH1*). Interestingly, all these HSPs have been found as present in oviductal extracellular vesicles (oEVs) at the protein and mRNA levels [94], and supplementation with oEVs during IVC improved embryo development and cryo-survival of embryos [95, 96]. We could hypothesize that the uptake of the EVs by the embryos may lead to an increase in HSP concentration in the embryo, helping it to recover from vitrification damages.

IPA analysis also pointed to Tretinoin (also known as retinol or vitamin A) as an upstream regulator of genes altered in VIT vs. IVC (also in VIT vs. CO) such as *TGFB1*, *FGF2* or *SAMD3*. Regulation of TGF–beta signaling by retinol has been reported extensively showing the ability to suppress or amplify TGF-beta signaling [97–99]. Given that retinol can negatively regulate *TGFB1*, we hypothesize that the addition of retinol to IVC or VIT media could decrease the expression of *TGFB1* which was strongly upregulated in VIT vs. IVC. The addition of retinol to IVM media in the pig improved the blastocyst rate and quality [100]. Similar results have been observed in cattle and rabbits [101, 102]. Even more, survival rates of cryopreserved blastocysts were improved when the oocyte received 9–cis–RA during pre–maturation [103]. These studies together with the present results point to retinol as a good supplement to prevent or overcome the VIT effects in porcine embryos.

## Conclusions

Our findings revealed specific alterations of the VIT procedure on the embryonic gene expression first by comparing differences in VIT vs. IVC embryos and second by an integrative analysis of three embryo comparisons (VIT, IVC, and CO), confirming the consistency of our findings. VIT-specific functional alterations were associated to response to osmotic stress, response to hormones, carbohydrate biosynthesis process, lipid transporter activity, and developmental growth. Moreover, our study provided functional alterations that might be related to the sum of VIT and IVC effects or only IVC effects such as response to hypoxia, mitophagy, and SLC mediator transporters. Overall, the VIT alterations reported here might reflect the transcriptional signature of the VIT cryodamage but also the VIT healing process of the embryos that successfully overcome the VIT process. Specific genes related to wound healing, tissue repair, cell proliferation, transport of small molecules and with known reproductive roles were pointed as potential biomarkers that may help to improve vitrification. These findings contribute to a better understanding of the VIT impact on pig embryos that can affect their development and fate. Moreover, it provides a strong molecular basis for further studies investigating intervention strategies.

## Supplementary Information

**Additional file 1: Table S1.** All identified transcripts in VIT vs. CO embryos.

**Additional file 2: Table S2.** All identified transcripts in IVC vs. CO embryos.

**Additional file 3: Table S3.** All identified transcripts in VIT vs. IVC embryos.

**Additional file 4: Table S4.** Differentially expressed genes (DEGs) in embryos for the VIT vs. CO comparison (FDR < 0.001). **Table S5.** Differentially expressed genes (DEGs) in embryos for the IVC vs. CO comparison (FDR < 0.001). **Table S6.** Differentially expressed genes (DEGs) in embryos for the VIT vs. IVC comparison (FDR < 0.001).

**Additional file 5: Table S7.** Differentially expressed genes (DEGs) in embryos for the VIT vs. CO comparison (FDR < 0.001 and log2 FC>2). **Table S8.** Differentially expressed genes (DEGs) in embryos for the IVC vs. CO comparison (FDR < 0.001 and log2 FC>2). **Table S9.** Differentially expressed genes (DEGs) in embryos for the VIT vs. IVC comparison (FDR < 0.001 and log2 FC>2).

**Additional file 6: Table S10.** Venn Diagram. The overlap of DEG among all based only on FDR < 0.001 and based on FDR < 0.001 and log2 FC>2. **Table S11.** List of gene comparisons among studies. **Table S12.** Proportions of genes up and down-regulated in the different embryo groups based on DEG and log 2 FC. **Table S13.** Ortholog annotation from Jan 2019 used in the study.

**Additional file 7.** Functional analysis of transcripts altered in vitrified, in vitro culture and control embryos. Contains: **Table S1.** Annotation of DEG in the three embryo comparisons: VIT vs. CO, IVC vs. CO and VIT vs. IVC. **Table S2.** Enrichment analysis of DEG in in the three embryo comparisons: VIT vs. CO, IVC vs. CO and VIT vs. IVC. **Table S3.** Membership data for embryo term by Metascape analysis. **Table S4.** Membership data for oxidative damage term by Metascape analysis. **Table S5.** Membership data for cell arrest term by Metascape analysis. **Table S6.** Membership data for stress response term by Metascape analysis. **Table S7.** List of genes derived from SOTA clustering analysis VIT vs. IVC comparison. **Table S8.** Functional analysis from cluster genes 1-4 derived from Metascape. **Table S9.** Enrichment annotation analysis from cluster genes 1-4 derived from Metascape. **Table S10.** Gene expression profile across samples for selected genes validated by qPCR.

## Data Availability

RNA–Seq data have been deposited at NCBI’s Sequence Read Archive (SRA) under the BioProject accession PRJNA697877 and is available at https://www.ncbi.nlm.nih.gov/sra/PRJNA697877. Additionally, all generated or analyzed data derived from this study are included in this published article and its Supplementary data files 1 and 2.

## Abbreviations

VIT: Vitrification
CPA: Cryoprotective agents
IVC: In vitro culture
CO: Fresh embryos
vs.: Versus
EG: Ethylene glycol
DMSO: Dimethyl sulfoxide
SOPS: Superfine open pulled straw
RNA: Ribonucleic acid
RIN: RNA integrity number
qPCR: Quantitative real–time polymerase chain reaction
EMP1: Epithelial membrane protein 1
ANXA8: Annexin A8
GSTA4: Glutathione s–transferase alpha 4
SERPINE1: Plasminogen activator inhibitor 1 precursor
PLET1: Placenta Expressed Transcript 1
BTG2: BTG anti-proliferation factor 2
SPP1: Secreted phosphoprotein 1
SLC10A1: Solute carrier family 10 member 1
FDR: False discovery rate
FC: Fold change
RNA–seq: RNA–sequencing
